# Adaptation and validation of the Diabetes Management Self-Efficacy Scale to Brazilian Portuguese^1^

**DOI:** 10.1590/1518-8345.1543.2861

**Published:** 2017-05-22

**Authors:** Ana Emilia Pace, Lilian Cristiane Gomes, Daniela Comelis Bertolin, Helena Maria Almeira Macedo Loureiro, Jaap Van Der Bijl, Lillie M. Shortridge-Baggett

**Affiliations:** 2 PhD, Associate Professor, Escola de Enfermagem de Ribeirão Preto, WHO Collaborating Centre for Nursing Research Development, Universidade de São Paulo, Ribeirão Preto, SP, Brazil.; 3 PhD, Professor, Centro Universitário da Fundação Educacional Guaxupé, Guaxupé, MG, Brazil.; 4 PhD, Professor, União das Faculdades dos Grandes Lagos, São José do Rio Preto, SP, Brazil.; 5 PhD, Professor, Escola Superior de Saúde da Universidade de Aveiro, Aveiro, Portugal.; 6 PhD, Researcher, Inholland University of Applied Sciences, Faculty of Health, Sports & Welfare, Amsterdam, Netherlands.; 7 EdD, Professor Emeritus, Lienhard School of Nursing, College of Health Professions, Pace University, New York, NY, USA.

**Keywords:** Self Efficacy, Diabetes Mellitus, Psychometrics, Nursing Assessment

## Abstract

**Objective:**

to perform the cultural adaptation and validation of the Diabetes Management Self-efficacy Scale for Patients with Type 2 Diabetes Mellitus with a Brazilian population sample.

**Method:**

cross-sectional methodological study in which the adaptation and validation process included the stages recommended in the literature. Construct validity and reliability were assessed with 200 adults with type 2 diabetes mellitus.

**Results:**

the items indicated by the panel of judges and by the target population were adjusted in the cultural adaptation to improve clarity and understanding. The instrument's four factors remained in the confirmatory factor analysis with factor loadings of items greater than 0.30, except for factor 4; convergent validity, verified by the multitrait-multimethod analysis, presented inter-item correlations from 0.37 to 0.92, while for discriminant validity, 100% of the items presented greater correlation in their own factors. Cronbach's coefficient alpha for the total scale was 0.78, ranging from 0.57 to 0.86 among factors.

**Conclusion:**

semantic, cultural, conceptual and idiomatic equivalences were achieved and the instrument's Brazilian version also presented psychometric properties that showed evidence of reliability and validity. Thus, it can be applied both in clinical practice and research. Self-efficacy is useful for planning and assessing educational interventions, as well as predicting behavior modification in self-care.

## Introduction

Self-efficacy (SE) is a key concept from Bandura's Social Cognitive Theory and is defined as one's perception of one's own ability to organize and perform actions(1). Beliefs regarding one's own ability vary in a given context or situation; i.e., beliefs are not uniform and are a result of personal experiences, others' verbal persuasion, social modeling through observation of other people's performances, and physical and emotional states. Therefore, SE is not a characteristic of one's personality nor does it reflect personal competencies themselves. Rather, it reflects one's belief or judgment regarding one's competencies(2).

The SE dimensions that influence human behavior include magnitude, strength and generality. Magnitude refers to one's perceived degree of difficulty to perform a given task; strength refers to one's personal conviction one is able to perform a specific task; and generality is seen as one's ability to extend SE from one situation to another(2).

In this study, SE is addressed in the context of care provided to individuals with diabetes mellitus (DM), for which a challenging regime is often imposed in which self-care behaviors range from the adoption of a healthy lifestyle, up to the handling of devices and inputs, to self-monitoring blood glucose levels and the administration of insulin(3). 

Self-care behavior requires personal judgments and decisions that tend to be difficult for individuals. These behaviors require technical and cognitive skills, which, in turn, are frequently associated with one's SE beliefs(4). SE, therefore, influences an individual's decisions regarding adopting or not adopting a given behavior, whether this individual will persevere in this behavior or not, and how well s/he will respond to obstacles and relapses of undesirable prior behaviors(5).

When DM education is considered as a strategy to develop self-care behavior, as well as to motivate its implementation and maintenance, SE is highlighted in the planning and assessment of educational interventions(6) and in predicting behavioral changes over time(7-8). Therefore, culturally adapted and validated instruments to assess SE need to be available both to be used in clinical practice and in research.

One of the instruments used to assess SE in the performance of behaviors aimed to control type 2 DM (DM2) is the Diabetes Management Self-Efficacy Scale for Patients with Type 2 Diabetes Mellitus (DMSES). Originally developed in the Netherlands(6), it was later adapted and validated for the Australian(9), Turkish(10), Chinese(11), and Arabic(12) contexts.

In Brazil, there are no valid instruments specifically to assess SE among individuals with DM2. Therefore, this study's objectives included the cultural adaptation and validation of the DMSES in a Brazilian population sample.

## Method

This methodological and cross-sectional study was conducted in an outpatient unit of a university hospital in the interior of São Paulo in two main stages: cultural adaptation and assessment of the psychometric properties of the adapted version, conducted from August 2009 to May 2012.

DMSES(6) is a Likert scale with 20 items distributed among four factors (1-Nutrition specific and weight; 2- Nutrition general and medical treatment; 3-Physical exercise; 4-Blood sugar), intended to assess SE in the practice of self-care behaviors to manage DM. These behaviors refer to three types of activities: activities that are essential to the treatment of diabetes (use of medication: oral anti-diabetics and/or insulin; dietary adherence; and exercise); self-observation activities (controlling/observing and recording blood sugar or sugar in the urine, body weight, the condition of feet skin, and general health condition); and self-regulating activities (correction of hypo- and/or hyperglycemia, preparation for vacation, diet changes, or self-regulation when there is weight gain, acute disease or stress)(6).

The scale's items express not only the behaviors individuals with DM are supposed to perform in each of the activities but also ask whether individuals feel capable of performing such behaviors. After a literature review and expert assessment, the study in which the original instrument was developed established that all items would begin with the expression "I think I'm able to" because this statement reflects "strength", which is considered the main component of SE(6). 

All the items present a response pattern from "definitely can do" up to "cannot do at all", with scores ranging from one to five, respectively. The instrument's global average score determines SE; that is, the score of each item is totaled and then divided by the number of items (20). High scores indicate high SE(6). 

The psychometric properties of the original instrument, which is available in English and Dutch, were assessed with a sample of 94 Dutch adults with DM2 and presented good internal consistency, the Cronbach's alpha of which was 0.81(6). The Australian version also showed internal consistency (α=0.91), though the authors recommend further psychometric tests to be applied with larger samples to check for potential redundancy of items(9).

The Cronbach's alpha of the Turkish version(10) was 0.88 and the exploratory factor analysis revealed three factors instead of the four presented by the original scale(6). The authors explain that cultural characteristics or the connotation of words in the target language may account for such a result.

The Cronbach's alpha found in the study in which the Chinese version was developed and adapted (C-DMSES)(11) was 0.93. The study also showed, through criterion validity, that the C-DMSES could predict self-care activities in a similar fashion to the Arabic version, the Cronbach's alpha of which was 0.91. The Arabic study showed that four out of its five domains predicted self-care behaviors(12). 

### Stage 1 - Adaptation of the instrument to the Brazilian culture

Permission was provided by the authors of the original version to translate the instrument into Brazilian Portuguese and the stages recommended for methodological studies(13-14) were followed, namely: translation to the target language; assessment of the translated version by a panel of judges; back translation so the authors could make a comparison with the original version; semantic analysis with the target population; pretest of the final Brazilian version with a small sample of individuals with similar characteristics of those of the target population; and psychometric analysis to validate the final translated version.

The first step was to choose the translators, which according to recommendations of experts in methodological studies(13-14), must be professionals who have mastered both English and culture and who present distinct profiles, that is, one translator who has knowledge of the concepts investigated by the instrument in order to obtain an equivalent translation from a clinical perspective and another translator who must have no knowledge in regard to these concepts so that the translation reflects the usual language of the target population. 

The translation was performed by two independent translators: a Brazilian teacher of English, with no knowledge of the topic, and a Belgian professional who translates papers in the health field and lives in Brazil. This stage resulted in two Portuguese versions, respectively named Portuguese version 1 (PV1) and Portuguese version 2 (PV2). Both translated versions were compared by the researchers and translators in order to select the best expressions and, as a result, a consensual Portuguese version was achieved (CPV1). 

The CPV1's semantic, idiomatic, conceptual, and cultural equivalence was assessed by a panel of judges. This panel was composed of four professionals from the health field with experience in care provided to DM patients and/or methodological studies, a bilingual translator and an individual affected by the disease with a college degree. The adjustments in the instrument were performed using a minimum interrater agreement of 80%(15), which resulted in the consensual Portuguese version 2 (CPV2). This assessment on the part of a panel of judges before the back translation identified potential errors or difficulties in understanding items, so that the instrument can be refined until the version that will be used with the target population is achieved(13-14).

The CPV2 was back translated by two bilingual independent translators, not the initial translators, with fluency in both English and Portuguese, in addition to having knowledge concerning Brazilian culture, who were not aware of the study's concepts(16). This stage was intended to assess whether the Portuguese version reflected the content of the original version in English. Two back translations were obtained (BT1 and BT2), which were analyzed together with the translators and researchers to reach a consensual version in English (CVE).

Afterwards, a semantic analysis using the CPV2 was conducted with 18 people of the target population, who were not included subsequently in the study sample. The participants' suggestions were incorporated into the instrument and the new version was once more submitted to the authors of the original version. The authors agreed with the changes and the final Brazilian version was obtained (FBV).

A pretest was applied with the FBV to assess the pertinence of items and the answer options. A form containing all the FBV's items, which were divided into subsets of items totaling five subsets with four items each, was applied. The participation of three individuals with DM2 was required for each subset, totaling 15 respondents. This form was based on studies conducted by the DISABKIDS group and addresses the relevance of each item and the difficulty answering each question, as well as whether the answer options were clear. If necessary, the respondent was allowed to rewrite the item in his/her own words(16).

### Stage 2 - Initial assessment of the adapted instrument's psychometric properties

The study's sample was composed of 200 adults with DM2, of both genders, receiving medical treatment with insulin and/or anti-diabetics, presenting no chronic complications in an advanced stage, recruited through their medical files on the day they attended return visits with healthcare professionals. The participants were verbally invited to participate in the study, received clarification regarding the study's objective, and read a free and informed consent form; those who consented signed the form. The sample size met the criterion of at least five and a maximum of ten respondents for each item in the instrument(17).

One of the researchers individually interviewed the patients, one time only, using the DMSES Brazilian version. Sociodemographic data were collected during the same opportunity. Data were typed and validated in MS-Excel and later transferred to SAS version 9.2, through which univariate and bivariate exploratory analysis were performed.

### Statistical Procedures

Confirmatory factor analysis, via structural equation modeling for latent variables, was used to test the hypothesis of the scale's factor composition, according to the four factors found in the literature. The model includes fixed parameters (factor loadings equal to zero) and free parameters to be estimated (factor loadings different from zero). Goodness of fit tests were performed to verify whether the factors explain the correlations among variables, according to the theoretical model proposed, described as follows. 

The Chi-square test was performed to verify goodness of fit (which checks whether the matrix of estimated covariance is equal to the sample's covariance matrix); the level of significance was established at p>0.05; for larger samples, this test is usually significant so that the use of χ2/DF is recommended; values lower than 2.0 indicate good fit; GFI (Goodness of Fit Index): acceptance region equal to or greater than 0.85; AGFI (GFI Adjusted for Degrees of Freedom): acceptance region equal to or greater than 0.80; SRMR (Standardized Root Mean Square Residual): acceptance region equal to or less than 0.10; RMSEA (Root Mean Square Error of Approximation): acceptance region equal to or less than 0.08; CFI (Bentler's Comparative Fit Index): acceptance region equal to or greater than 0.90; NNFI (Bentler & Bonett's Non-Normed Fit Index): acceptance region equal to or greater than 0.90.

Analysis of data's goodness of fit for the proposed factors was performed using significance tests for factor loadings (t>1.96 indicates the item has significant loading within the factor) and for the proposition of factor modifications, by excluding items of certain factors, the Wald test was used to verify the extent the withdrawal of an item would influence a decrease in the model's Chi-square statistics. If this change is not significant, the item can be withdrawn without affecting future results. The Lagrange multiplier test, which establishes the need to relocate an item to another factor, was also performed to improve the correlation among the items in the same factor. Similar to the Wald test, it shows the extent to which Chi-square statistics will change if an item is relocated to another factor.

Construct validity was then performed by means of convergent and discriminant validity obtained through multitrait-multimethod matrix (MTMM), which describes the magnitude of correlations between items and factors. Convergent validity verified in initial validation studies is satisfied when the linear correlation between an item and the factor to which it belongs equals 0.30. For discriminant validity, the linear correlation between an item and its factor is expected to be greater than its correlation to other factors in most correlations considered(17).

Reliability was estimated by Cronbach's alpha and by the correlation of each factor with the total scale. The level of significance adopted was 5%. 

### Ethical aspects

This study was approved by the Institutional Review Board at the Hospital das Clínicas, Ribeirão Preto School of Medicine, University of São Paulo (HCFMRP-USP), under Report No. 2889/2006.

## Results

### Panel of judges' analysis: semantic, idiomatic, conceptual and cultural equivalences

The analysis performed by the panel of judges required a few changes in the title, instructions and items. The title in Portuguese was Escala de Autoeficácia no "Gerenciamento" de Diabetes para Pacientes com Diabetes Mellitus tipo 2 [Self-Efficacy Scale for the "Management" of Diabetes for Patients with Type 2 Diabetes Mellitus] and was changed to Escala de Autoeficácia no "Controle" do Diabetes para Pacientes com Diabetes Mellitus tipo 2 [Self-Efficacy Scale for the "Control" of Diabetes for Patients with Type 2 Diabetes Mellitus]. The first instruction concerning how to complete the instrument was Por favor, responda cada pergunta marcando a resposta que descreve o quanto você sente-se capaz de "cuidar" do seu diabetes" [Please answer each question choosing the answer that describes how able you feel to "care for" your diabetes], which became Por favor, responda cada pergunta marcando a resposta que descreve o quanto você sente-se capaz de "controlar" o seu diabetes [Please answer each question choosing the answer that describes how able you feel to "control" your diabetes] and the second instruction was Por favor, responda às duas próximas perguntas se você toma medicamentos (comprimidos) para o seu diabetes [Please answer the next two questions if you take medication (pills) for your diabetes], which became Por favor, responda às duas próximas perguntas se você toma medicamentos (comprimidos/insulina) para o seu diabetes [Please answer the next two questions if you take medication (pills/insulin) for your diabetes].

In regard to the items, some adjustments were made in terms of phrasing and wording. In the questions that contained the phrases para o diabetes [for the diabetes] and quando eu estiver [when I am], were replaced with para o controle do diabetes [to control the diabetes] and quando eu estou [when I am] ; the phrases descobrir problemas na pele [discover skin problems] was replaced with ver se tenho problemas na pele [see if I have skin problems]; quando eu estiver com estresse ou tensão [when I have stress or tension] by quando estou estressado ou tenso [when I am stressed or tense]; and uma vez por ano [once a year] with regularmente [regularly], and atividades físicas [physical activities] with exercícios físicos [physical exercises]. The replaced words were: aconselhar [counseling] with recomendar [recommending] and prescrição [prescription] with receita médica [medical prescription]. Additionally, the personal pronoun "I" was suppressed from the middle of sentences and articles before possessive pronouns were deleted. 

### Semantic analysis of items performed by the target population

The semantic analysis of items performed with the CPV2 by 18 individuals with DM2 revealed difficulties understanding item 1 (Eu acho que sou capaz de verificar meu açúcar no sangue, se necessário [I think I'm able to check my blood sugar, if necessary]). Many of the respondents did not have a glucose meter and, for this reason, did not check their capillary glycemia as part of their routine. Nonetheless, this item was kept because this was a specific understanding problem and the recommendation is to encourage people to self-monitor their glucose levels at home.

The respondents also showed problems understanding items 9 (Eu acho que sou capaz de ajustar a minha dieta quando estou doente) [I think I am able to adjust my diet when I am sick] and 20 (Eu acho que sou capaz de ajustar meus medicamentos quando estou doente) [I think I am able to adjust my medication when I am sick] because in the phrase "when I am sick" the respondents understood "sick" as being sick with DM rather than with some other condition, such as an infection or an acute disease. Therefore, we decided to add examples of other diseases at the end of these items such as the flu, cold or infection, to improve understanding.

Suggestions were also proposed by the respondents for items 16 (Eu acho que sou capaz de seguir minha dieta quando estou numa festa) [I think I am able to follow my diet when I am at a party] and 18 (Eu acho que sou capaz de ir ao médico regularmente para controlar o meu diabetes) [I think I am able to go to the doctor regularly to control my diabetes], which respectively resulted in Eu acho que sou capaz de seguir a minha dieta quando estou numa comemoração/festa [I think I am able to follow my diet when I am at a celebration/party] and Eu acho que sou capaz de ir ao médico regularmente para acompanhar o meu diabetes [I think I am able to go to the doctor regularly to monitor my diabetes].

At the end of this stage, the difficulties had been identified and the respondents' suggestions were sent via email to the instrument's authors, who accepted the solutions as being pertinent. Afterwards, we assessed the psychometric properties of the FBV, which was then named Escala de autoeficácia no controle do diabetes para pacientes com Diabetes Mellitus tipo 2 [Self-Efficacy Scale for the Control of Diabetes for Patients with Type 2 Diabetes Mellitus].

### Pretest of the final Brazilian version (FBV)

Assessments concerning the relevance of each item, difficulties answering them, and options for answers were considered satisfactory by those who took part in the pretest, who also agreed with the construction of the items. The individuals in the target population did not manifest the need to make any changes in the redaction or how the items are presented, thus, the instrument was considered easily understood and accepted. Therefore, this stage of adaptation was considered to be complete and we then proceeded to validate the translated instrument.

### Psychometric properties of the Brazilian Version of the Diabetes Management Self-efficacy Scale for Patients with Type 2 Diabetes Mellitus

The assessment of psychometric properties was conducted with a sample of 200 individuals with DM2. The interviews lasted 40 minutes on average. The sample's sociodemographic characterization showed that the participants were aged 60 years old on average (SD=9.63). Of the 200 participants, 111 (55.5%) were women; 128 (64.3%) were married/in a stable relationship; and 96 were retired/pensioners (48%), followed by those performing non-paid household work (26.5%). Years of education and time since diagnosis were five years (SD=3.74) and 15.7 years (SD=8.36) on average, respectively.

The total average score for the SE scale was 4.05 (SD=0.58), according to an interval ranging from 1 to 5. The item with the lowest average was item 16 Eu acho que sou capaz de seguir minha dieta, quando estou numa comemoração/festa [I think I am able to follow my diet when I am at a celebration/party] and the item with the highest average was item 18 Eu acho que sou capaz de ir ao médico regularmente para acompanhar o meu diabetes [I think I am able to go to the doctor regularly to monitor my diabetes] (Table 1).

**Table 1:** Description of items and the final score of the Brazilian Version of the Diabetes Management Self-efficacy Scale for Patients with Type 2 Diabetes Mellitus. Ribeirão Preto, SP, Brazil, 2012

Items (N=200)	Average (SD*)
1. Eu acho que sou capaz de verificar meu açúcar no sangue, se necessário	4.44 (1.27)
2. Eu acho que sou capaz de corrigir meu açúcar no sangue, quando o valor estiver muito alto	4.25 (1.25)
3. Eu acho que sou capaz de corrigir meu açúcar no sangue, quando o valor estiver muito baixo	4.48 (1.07)
4. Eu acho que sou capaz de escolher os alimentos certos para o controle do diabetes	4.27 (1.10)
5. Eu acho que sou capaz de escolher alimentos diferentes, sem sair da dieta recomendada para o controle do diabetes	3.82 (1.36)
6. Eu acho que sou capaz de manter o meu peso sob controle	3.62 (1.58)
7. Eu acho que sou capaz de examinar meus pés para ver se tenho problemas na pele	4.49 (1.10)
8. Eu acho que sou capaz de fazer exercícios físicos suficientes para o controle do diabetes, por exemplo, caminhar ou andar de bicicleta	3.49 (1.75)
9. Eu acho que sou capaz de ajustar a minha dieta quando estou doente, como, por exemplo, gripe, resfriado ou infecção	4.21 (1.25)
10. Eu acho que sou capaz de seguir a minha dieta a maior parte do tempo	4.23 (1.25)
11. Eu acho que sou capaz de fazer exercícios físicos extras, quando o médico recomendar	3.40 (1.67)
12. Eu acho que sou capaz de ajustar a minha dieta, quando faço exercícios físicos extras	3.52 (1.51)
13. Eu acho que sou capaz de seguir minha dieta, quando estou fora de casa	3.77 (1.43)
14. Eu acho que sou capaz de ajustar minha dieta, quando estou fora de casa	3.75 (1.41)
15. Eu acho que sou capaz de seguir minha dieta, quando estou de férias	4.07 (1.21)
16. Eu acho que sou capaz de seguir minha dieta, quando estou numa comemoração/festa	3.39 (1.62)
17. Eu acho que sou capaz de ajustar minha dieta, quando estou estressado ou tenso	3.52 (1.61)
18. Eu acho que sou capaz de ir ao médico regularmente para acompanhar o meu diabetes	4.89 (0.45)
19. Eu acho que sou capaz de tomar meus medicamentos, de acordo com a receita médica	4.84 (0.55)
20. Eu acho que sou capaz de ajustar meus medicamentos, quando estou doente, como, por exemplo, gripe, resfriado ou infecção	4.49 (1.11)
Total	4.05 (0.58)

*Standard Deviation

### Analysis of validity

The four factors proposed in the development of the original instrument remained in the Confirmatory Factor Analysis (CFA)(6): Factor 1, Specific nutrition and weight (items 6, 13, 14, 15 and 16); Factor 2, General nutrition and medical treatment (items 4, 5, 7, 9, 10, 17, 18, 19 and 20); Factor 3, Physical exercise (items 8, 11 and 12); and Factor 4, Blood sugar (items 1, 2 and 3). Additionally, goodness of fit was acceptable according to the proposed theoretical model (Standardized Root Mean Square Residual-SRMR) (Table 2). 

By estimating factor loading, we verified that most items obtained significant loading in their factors, except factor 4 (Table 3). The exclusion of items 1, 2 and 3 and the relocation of item 15 to factor 4 was suggested.

**Table 2:** Goodness of fit statistics according to the confirmatory factor analysis of the self-efficacy scale*. Ribeirão Preto, SP, Brazil, 2012

Goodness of fit statistics	Values
Chi-Square	419.17
Chi-Square DF	164
P-value	<0.00
Chi-Square Ratio	2.55
Goodness of Fit Index (GFI)	0.82
GFI Adjusted for Degrees of Freedom (AGFI)	0.77
Bentler's Comparative Fit Index	0.74
Bentler & Bonett's (1980) NNFI	0.65
Standardized Root Mean Square Residual (SRMR|)	1.00
RMSEA Estimate† - Root Mean Square Error of Approximation	0.09

*Number of items in the scale: 20 items; n=200 participants. Acceptable values: P-value Chi-Square>0.05; Chi-square ratio <2.0; GFI≥0.85; AGFI≥0.80; SRMR≤0.10; RMSEA≤0.08; CFI≥0.90; NNFI≥0.90; † RMSEA - Root Mean Square Error of Approximation.

**Table 3:** Factor loading of the items in the Brazilian Version of the Diabetes Management Self-efficacy Scale for Patients with Type 2 Diabetes Mellitus, obtained through confirmatory factor analysis. Ribeirão Preto, SP, Brazil, 2012

Factor 1 Items	Load	Value of t	Factor 2 Items	Load	Value of t	Factor 3 Items	Load	Value of t	Factor 4 Items	Load	Value of t
6	0.30	3.98*	4	0.48	6.53*	8	0.80	12.86*	1	0.00	0.00
13	0.82	12.34*	5	0.46	6.13*	11	0.93	15.68*	2	-0.01	-0.00
14	0.74	10.99*	7	0.19	2.42*	12	0.76	11.97*	3	-0.00	-0.00
15	0.52	7.12*	9	0.53	7.27*						
16	0.53	7.32*	10	0.57	7.89*						
			17	0.45	6.02*						
			18	0.35	4.61*						
			19	0.37	4.87*						
			20	0.35	4.66*						

*Significant values of the item's standardized loadings (p<0.05) for t>1.96.

The CFA was recalculated considering the suggested exclusions and relocation of items. Some goodness of fit criteria obtained values that were acceptable according to the theoretical model (GFI, AGFI, SRMR and RMSEA). The estimation of factor loadings showed that all items presented significant loadings in their factors and no exclusion of items was suggested, though it was suggested that item 7 be relocated to factor 3 (data not shown).

After analysis concerning the second change, only the relocation of item 6 to factor 2 was suggested; however, we decided to cease analysis considering the few changes observed in the goodness of fit statistics.

The MTMM analysis performed between the items and their respective factors showed convergent and discriminant validity. Inter-item correlations ranged from 0.37 to 0.92 with an average of 0.64, while 100% of the items presented higher correlations within their own factors (Table 4).

**Table 4:** Correlations between items and factors according to the confirmatory factor analysis of the Brazilian Version of the Diabetes Management Self-efficacy Scale for Patients with Type 2 Diabetes Mellitus, Ribeirão Preto, SP, Brazil, 2012

Factors	01			02			03			04	
Items*	r†	p-value‡		r†	p- value‡		r†	p- value‡		r†	p- value‡
06	0.54	<0.0001		0.29	<0.0001		0.22	0.001		-0.12	0.10
13	0.79	<0.0001		0.38	<0.0001		0.09	0.21		0.01	0.84
14	0.73	<0.0001		0.37	<0.0001		0.11	0.13		0.003	0.96
15	0.63	<0.0001		0.44	<0.0001		0.23	0.001		0.002	1.00
16	0.70	<0.0001		0.34	<0.0001		0.12	0.09		-0.06	0.41
04	0.33	<0.0001		0.54	<0.0001		0.11	0.13		0.17	0.01
05	0.26	0.0002		0.58	<0.0001		0.26	0.0002		0.13	0.07
07	0.03	0.66		0.37	<0.0001		0.28	<0.0001		0.05	0.45
09	0.31	<0.0001		0.58	<0.0001		0.08	0.29		0.20	0.005
10	0.50	<0.0001		0.67	<0.0001		0.23	0.0008		-0.03	0.65
17	0.48	<0.0001		0.56	<0.0001		0.16	0.02		0.01	0.86
18	0.12	0.10		0.37	<0.0001		0.06	0.37		0.20	0.004
19	0.13	0.06		0.37	<0.0001		0.11	0.11		0.32	<0.0001
20	0.05	0.52		0.44	<0.0001		0.12	0.10		0.21	0.003
08	0.20	0.004		0.28	<0.0001		0.89	<0.0001		0.01	0.90
11	0.21	0.003		0.26	0.002		0.92	<0.0001		0.06	0.38
12	0.19	0.01		0.32	<0.0001		0.85	<0.0001		0.05	0.50
01	-0.19	0.01		-0.13	0.07		-0.06	0.38		0.72	<0.0001
02	0.03	0.67		0.39	<0.0001		0.11	0.11		0.78	<0.0001
03	0.06	0.41		0.25	0.0004		0.05	0.49		0.70	<0.0001

*Number of items in the scale: 20 items; n=200 participants; †r = Pearson's coefficient of correlation; ‡Significant values (p<0.05)

### Reliability analysis

The internal consistency of the four domains, calculated by Cronbach's alpha, was 0.78 for the total scale. Note that higher values were found for factors 1 and 3, and moderate values were found for the remaining factors (Table 5).

**Table 5:** Analysis of total internal consistency and of the four factors of the Brazilian Version of the Diabetes Management Self-efficacy Scale for Patients with Type 2 Diabetes Mellitus. Ribeirão Preto, SP, 2012

Factors (Items)	No. de Items	Cronbach's alpha (α)	Items with lower consistency	Correlation Item/ Total*	Cronbach's alpha if item was excluded †
Factor 1 (13, 14, 15, 16, 6)	5	0.71	---	---	---
Factor 2 (5, 10, 9,7, 18, 4, 19, 17, 20)	9	0.64	SE‡7	0.17	0.65
Factor 3 (11, 12, 8)	3	0.86	---	---	---
Factor 4 (2, 3, 1)	3	0.57	---	---	---
Total / Orthogonal	20	0.78	SE‡1	-0.08	0.80

*Correlation of item with total of the respective domain, not considering the item in the total score.

† Cronbach's α after removing items with lower consistency.

‡SE - self-efficacy

## Discussion

The assessment of the first consensual version in Brazilian Portuguese (CVP1) of the DMSES by the panel of judges enabled refining the instrument's semantic, idiomatic, conceptual and cultural equivalences and a second consensual version in Portuguese (CVP2) was then obtained. The following stage (back translation) helped to verify the pertinence of this version, leading to semantic analysis with the target population. 

The items identified in the semantic analysis as being unclear were revised and discussed with the instrument's authors. Item 1 Eu acho que sou capaz de verificar meu açúcar no sangue, se necessário [I think I am able to check my blood sugar, if necessary], was kept because, even though SE is related to one's perception of one's ability at the present moment, people can easily imagine themselves to be able or unable to perform a task when considering a hypothetical situation(1). 

Note that the self-monitoring of blood glucose demands visual acuity, and manual and cognitive skills, in addition to financial resources, which represent a barrier to many people, especially older individuals and those with low levels of education or income(18). Therefore, lack of understanding of this item may be related to procedural issues of self-monitoring. This result is similar to that found in the case of the Arabic version, in which the respondents had difficulties in regard to items related to the self-monitoring of blood glucose because they do not have the inputs necessary in their homes, and, for this reason, such a practice was not part of their routines(12).

The suggestion to add the word "celebration" to item 16 was considered pertinent because, in the Brazilian culture, not only parties, but also celebrations of all sorts involve the consumption of food and drinks. Eating is a social activity, the symbolic functions of which permeate interpersonal relationships and reveal the structure of daily life, satisfying not only a biological need but a social function, as well(19).

In regard to item 18, the term "monitor" was considered more pertinent than "control", especially because, in the context of an individual with a chronic disease, the control of DM depends more on the individual him/herself than on healthcare personnel(17). The decisions made by an individual with DM to control the disease have a greater impact on their wellbeing than those made by professionals(20). Healthcare practitioners should remain spectators, monitoring and providing guidance to empower chronic patients(21).

Some sociodemographic characteristics stand out in the validation process: a higher number of women, adults, low educational levels, and retired individuals, characteristics that are similar to those reported by studies concerning the original version(6), as well as the Australian(9), Turkish(10), and Chinese(11) versions. 

Descriptive analysis of the scale revealed that the items with the lowest and highest averages were respectively items 16 and 18. One descriptive study with a qualitative approach conducted with 24 Brazilian adults with DM, which aimed to identify difficulties concerning the treatment of the disease, reports that one of the main difficulties for these individuals involves following their diets, especially during social events, revealing that breaking a diet and the desire for foods are common circumstances in the lives of people with DM(22). 

On the other hand, the high SE verified in this study, regarding being able to attend medical return visits to monitor the disease, is similar to the results reported by descriptive Brazilian studies addressing individuals with DM and/or hypertension(21,23), which show the extent to which medical care is valued in the Brazilian culture(11). One cross-sectional study designed to assess the relationships among SE, self-care and the glycemic control of Jordanian adults with DM2, using the Arabic version of DMSES, also reports that the highest average was found for the item concerning medical follow-up(11). 

The results concerning the descriptive analysis of items reinforce the belief that SE can vary among self-care behaviors(12), that is, an individual may consider him/herself able to attend medical visits to monitor the disease but not able to follow a diet regime. Each behavior, in turn, may require different skills and types of knowledge, in addition to different levels of motivation and confidence(5,12).

The distribution of items in the factors according to the CFA of the Brazilian version remained similar to the original version(6). In the first phase of the CFA, however, three items (1, 2 and 3) that compose factor 4 (blood sugar) were indicated for exclusion, in addition to item 15. 

The DMSES proposes the assessment of SE in the performance of self-care behaviors concerning three types of activities, respectively named "activities that are essential for the treatment of diabetes", "self-observation activities" and "self-regulating activities"(6). Items 1, 2 and 3 of factor 4 refer to one's belief in one's ability to self-monitor and correct blood sugar and correspond to "self-observation activities" and "self-regulation"(6). Therefore, the exclusion of these items in this stage of validation could nullify central aspects of the instrument and hinder global understanding of the SE construct, in addition to the de-characterization of specific characteristics of the studied sample that would possibly interfere in these self-care activities. 

Correcting blood sugar by changing dosages of medications is not a common practice among the people with DM2 addressed in this study, due to a lack of knowledge and skills (data not shown). One descriptive study conducted in the same setting as this study with a sample of adults with DM2 showed that, among self-care activities, blood sugar monitoring was performed with a frequency below what is recommended(24).

Therefore, factor 4 was kept with its respective items, in accordance with the proposal by the authors of the original scale. We suggest that future studies be developed with samples from different Brazilian regions, or even with different sociodemographic and clinical profiles, to revalidate the instrument's psychometric properties, in addition to intervention studies to promote the knowledge and skills necessary for self-care and consequent improvement of SE, especially activities concerning self-monitoring of blood sugar.

The CFA indicated the need to relocate item 15 (Eu acho que sou capaz de seguir minha dieta, quando estou de férias) [I think I am able to follow my diet when I am on vacation] from Factor 1 (Specific nutrition and weight) to factor 4 (Blood sugar). Note this item can express "activities that are essential for the treatment of the disease" and "self-regulating activities", and due to the reasons already set out to keep items 1, 2 and 3 in factor 4, we also decided to keep item 15 in factor 1, also considering the coherence existing among the remaining items that compose this factor. 

Exploratory factor analysis was conducted for the study that validated the Chinese version of DMSES, which addressed a sample of 230 adults with DM2 in follow-up in an outpatient clinic in Taiwan, and four factors were also obtained. These factors, however, were named and composed as follows: nutrition (items 4, 5, 9, 10, 13, 14, 15, 16 and 17); verification activities (items 1, 2, 3 and 7); physical exercise and weight (items 6, 8, 11 and 12); and medical treatment (items 18, 19 and 20)(11). 

In contrast with the original and Brazilian version of DMSES, the study in which the Turkish version was adapted and validated using a sample of 110 adult individuals with DM2 in follow-up in the outpatient clinic of a university hospital found three factors: 1- nutrition and feet inspection; 2- medical control/treatment; and 3- physical exercise(10). Variations in the distribution of items may be related to cultural factors or to the sample size or clinical characteristics(10-11).

In regard to the construct validity, the results of the MTMM analysis were satisfactory in establishing convergent validity with correlations that ranged from 0.37 to 0.92 with an average of 0.64, values that are greater than the minimum values recommended by the literature (0.30). The results concerning discriminant validity reinforce the instrument's structure in which four factors should discriminate among SE beliefs regarding DM control in different aspects, such as Specific nutrition and weight; General nutrition and medical treatment; Physical exercise; and Blood sugar.

The convergent validity of the Australian(9) and Chinese(11) versions of DMSES were verified through linear correlations with the instrument General Self-Efficacy Scale (GSE), developed in Germany and adapted to 28 languages. Correlations of 0.52 (p<0.001) and 0.55 (p<0.01) were found for the Australian and Chinese versions, respectively, and confirmed the validity of these two versions to assess SE. Note that this modality of analysis was not performed in this study because we did not identify any similar instruments validated for our target population (individuals with DM2).

The analysis of reliability for the Brazilian version resulted in a Cronbach's coefficient alpha for the total scale of 0.78. The lowest alpha, 0.57, was found for factor 4 and the highest was 0.86 for factor 3. High internal consistency was verified for factors 1 and 3 and moderate consistency was found for the remaining factors. A review study addressing the psychometric properties of instruments aiming to assess subjective phenomena reports that values above 0.50 are consider reasonable(25).

The original version presented a Cronbach's alpha of 0.81(25) for the total scale and the lowest and highest values were 0.71 and 0.79, respectively. In contrast with this study, the original version's factors 1 and 2(6) presented the highest internal consistency.

The Cronbach's alphas of the Australian (n= 88)(9), Turkish (n=110)(10), and Chinese (n= 230)(11) versions of DMSES were 0.91, 0.88 and 0.93, respectively, similar to the coefficients found in the original instrument and in this study.

The fact that a non-probabilistic sample was used constitutes a limitation because the results obtained for the instrument's adaptation and validation cannot be generalized to other samples, especially considering Brazil's social and cultural diversity and clinical characteristics that differentiate the levels of complexity in care delivery.

## Conclusion

The process of adapting and validating the Escala de autoeficácia no controle do diabetes para pacientes com diabetes mellitus tipo 2 [Self-Efficacy Scale for the "Control" of Diabetes for Patients with Type 2 Diabetes Mellitus], for the Brazilian context followed the stages recommended in the literature. Idiomatic, semantic, cultural and conceptual equivalence was established with the original version, and psychometric properties provided evidence of the instrument's reliability and validity. 

Both in terms of clinical practice and research in the field of nursing, self-efficacy in the control of diabetes mellitus has been addressed in the planning of care and assessment of educational interventions. Therefore, we stress the importance of studies providing culturally adapted and valid instruments to assess self-efficacy and contribute to the advancement of knowledge concerning the care provided to individuals with this form of diabetes.

## References

[1] Bandura A (1977). Social Learning Theory.

[2] Bandura A (2012). On the Functional Properties of Perceived Self-Efficacy
Revisited. J Manage.

[3] Prado MD, Soares DA (2015). Limites e estratégias de profissionais de saúde na adesão ao
tratamento do diabetes: revisão integrativa. Rev Pesqui Cuidado Fundamental Online.

[4] Pereira MG, Almeida P (2004). Auto-eficácia na diabetes: conceito e validação da
escala. Anál Psicol.

[5] Beckerle CM, Lavin MA (2013). Association of self-efficacy and self-care with glycemic control in
diabetes. Diabetes Spectr.

[6] Van der Bijl JJ, Van Poelgeest-Eeltink A, Shortridge-Bagget L (1999). The psychometric properties of the diabetes management self-efficacy
scale for patients with type 2 diabete mellitus. J Adv Nurs.

[7] Frei A, Svarin A, Steurer-Stey C, Puhan MA (2009). Self-efficacy instruments for patients with chronic diseases suffer
from methodological limitations - a systematic review. Health Qual Life Outcomes.

[8] Mishali M, Omer H, Heymann AD (2011). The importance of measuring self-efficacy in patients with
diabetes. Family Practice.

[9] McDowell J, Courtney M, Edwards H, Shortridge-Bagget L (2005). Validation of the Australian/English version of the Diabetes
Management Self-Efficacy Scale. Int J Nurs Pract.

[10] Kara M, Van der Bijl JJ, Shortridge-Bagget L, Asti T, Erguney S (2006). Cross-cultural adaptation of the diabetes management self-efficacy
scale for patients with type 2 diabetes mellitus: Scale
development. Int J Nurs Stud.

[11] Wu SV, Courtney M, Edwards H, McDowell J, Shortridge-Bagget L, Chang P (2008). Development and validation of the Chinese version of the Diabetes
Management Self-efficacy Scale. Int J Nurs Stud.

[12] Al-Khawaldeh AO, Al-Hassan MA, Froelicher ES (2012). Self-efficacy, self-management, and glycemic control in adults with
type 2 diabetes mellitus. J Diabetes Complicat.

[13] Beaton D, Bombardier C, Guillemin F, Ferraz MB (2002). Recommendations for the cross-cultural adaptation of health status
measures.

[14] Freitas NO, Caltran M, Dantas RAS, Rossi LA (2014). Translation and cultural adaption of the Perceived Stigmatization
Questionnaire for burn victims in Brazil. Rev Esc Enferm USP.

[15] Medeiros RKS, MA Ferreira, Pinto DPSR, Vitor AF, Santos VEP, Barichello E (2015). Modelo de validação de conteúdo de Pasquali nas pesquisas em
Enfermagem. Rev Enferm Referência.

[16] Deon KC, Santos DMSS, Reis RA, Fegadolli C, Bullinger M, Santos CB (2011). Translation and cultural adaption of the Brazilian version of
Disabkids(r) Atopic Dermatits Module (ADM).. Rev Esc Enferm USP.

[17] Pasquali L (2003). Teoria dos testes na psicologia e na educação.

[18] Teixeira CRS, Zanetti ML, Landim CAP, Becker TAC, Santos ECB, Franco RC (2009). Automonitorização da glicemia capilar no domicílio: revisão
integrativa da literatura. Rev Eletron Enferm.

[19] Moreira SA (2010). Alimentação e comensalidade: aspectos históricos e
antropológicos. Cienc Cult.

[20] Funnell MM, Anderson RM (2004). Empowerment and self-management of diabetes. Clin Diabet.

[21] Taddeo PS, Gomes KWL, Caprara A, Gomes AMA, Oliveira GC, Moreira TMM (2012). Acesso, prática educativa e empoderamento de pacientes com doenças
crônicas. Cienc Saúde Coletiva.

[22] Péres DS, Santos MA, Zanetti ML, Ferronato AA (2007). Difficulties of diabetic patients in the illness control: feelings and
behaviors. Rev. Latino-Am. Enfermagem.

[23] Araújo JC, Guimarães AC (2007). Controle da hipertensão arterial em uma unidade de saúde da
família. Rev Saúde Pública.

[24] Coelho ACM, Gomes-Villas Boas LC, Gomides DS, Foss-Freitas MC, Pace AE (2015). Atividades de autocuidado e suas relações com controle metabólico e
clínico das pessoas com diabetes Mellitus. Texto Contexto Enferm.

[25] Mota DDCF, Pimenta CAM (2007). Avaliação e mensuração de variáveis psicossociais: desafio para
pesquisa e clínica de enfermagem. Rev Gaúcha Enferm.

